# Nursing homes underreport antipsychotic use but overreport diagnoses qualifying for appropriate use

**DOI:** 10.1093/haschl/qxad022

**Published:** 2023-06-20

**Authors:** Amanda C Chen, David C Grabowski

**Affiliations:** Harvard Graduate School of Arts and Sciences, Cambridge, MA 02138, United States; Department of Health Care Policy, Harvard Medical School, Boston, MA 02115, United States

**Keywords:** quality reporting, nursing homes, long-stay residents, antipsychotic

## Abstract

Antipsychotic drug use in US nursing homes remains a priority concern, but less is understood about the characteristics associated with reporting. Using linked Medicare claims and Minimum Data Set (MDS) assessments for long-stay nursing home residents from January 2018 to December 2019, we assessed the consistency of antipsychotic drug reporting and diagnosis of conditions (schizophrenia, Tourette's syndrome, and Huntington's disease) that qualify as appropriate drug use across data sources by calculating reporting rates in facility-reported MDS and Medicare claims. The antipsychotic reporting outcome is conditional on claims reporting while the condition reporting outcomes are conditional on MDS reporting. We found underreporting (87% reporting rate) in facility-reported antipsychotic use versus Medicare claims. In contrast, we found overreporting of the qualifying conditions with several facility-reported diagnoses unsupported by a corresponding claims diagnosis. Only 54.8% of schizophrenia, 46.5% of Tourette's syndrome, and 72.4% of Huntington's disease diagnoses reported in the MDS had a claims diagnosis. There was also variation in reporting odds for antipsychotic drug use by dual-eligibility status and race, with higher odds for dual-eligible and lower odds for Black residents. These findings suggest that CMS should continue investigating the source of reporting discrepancies in antipsychotic drug use and qualifying diagnoses.

## Introduction

The overprescription of antipsychotic drugs for US nursing home residents has been a persistent concern for decades and remains a priority issue for the Centers for Medicare and Medicaid Services (CMS). In particular, there has been concern that antipsychotics are used off-label in residents with dementia to control their behavioral and psychological symptoms, including aggression, agitation, irritability, and wandering.^1^ In response, CMS launched the National Partnership to Improve Dementia Care in Nursing Homes in 2012 to reduce antipsychotic drug use through stakeholder engagement, educational resources, public reporting, training of state surveyors, and increased regulatory enforcement. In the 5 years after the launch of the National Partnership, the prevalence of potentially inappropriate antipsychotic use among long-term nursing home residents decreased by 33%.^2^

One component of the National Partnership included public reporting of antipsychotic drug use for each nursing home on the Care Compare website, beginning in 2012.^3^ The purpose of the Nursing Home Compare and the Five-Star Quality Rating System is to provide summary measures of quality (from 1 to 5 stars) to inform nursing home choice and drive quality improvement.^4^ The percentage of residents newly prescribed or currently receiving antipsychotics was added to the Five-Star Quality Rating System on Care Compare in 2015.^2^ This measure treats the use of antipsychotics for residents with schizophrenia, Tourette's syndrome, or Huntington's disease (referred to as “qualifying conditions”) as “appropriate.” As such, residents with these qualifying conditions are excluded from the quality measure calculation to not penalize facilities for caring for these resident populations. This contrasts with “potentially inappropriate” use, which refers to antipsychotic drug use among residents without one of the qualifying conditions. The data source for the Five-Star Quality Rating System draws from the Minimum Data Set (MDS), which contains facility-reported information on resident assessments.

Despite the success of the National Partnership, there remains uncertainty about antipsychotic drug use reported by nursing homes. Because MDS assessment information is self-reported, there are concerns about discrepancies in information reported in the MDS compared with Medicare billing claims.^5,6^ Prior research established a low correlation between adverse events reported by nursing homes in the MDS and Medicare claims, including reporting rates of 58% for falls^7^ and 60% for pressure ulcers among long-stay residents, with higher rates among Black residents compared with White residents.^8^

More recent work by the Office of Inspector General (OIG)^9^ found that the MDS provided an incomplete assessment of the number of residents receiving antipsychotic drugs. Specifically, 5% of Medicare Part D long-stay residents in 2018 had a Part D claim for an antipsychotic drug but were not reported in the MDS as receiving the drug. Other work identified that nursing homes underreported their antipsychotic use by 1 percentage point per quarter relative to Part D claims, with this gap increasing after the Partnership in 2012.^10^ A 2022 OIG follow-up report^11^ studying the long-term trends of psychotropic drug use, which included antipsychotics as well as anticonvulsants and mood stabilizers, found that 80% of long-stay residents were prescribed a psychotropic drug. Psychotropic drug use was greater in nursing homes with lower registered nurse staffing ratios and higher shares of residents receiving Medicare Part D low-income subsidies (LIS).

In addition to concerns about the underreporting of antipsychotic use in nursing homes, there are questions about whether patients acquire diagnoses in the MDS, as the rate of residents reported with schizophrenia in the MDS have increased.^6,11^ This phenomenon arises when a resident does not have a corresponding Medicare claims diagnosis or medical documentation to support the diagnosis of the condition in the MDS (so called “phony diagnoses”).^6^

The purpose of this cross-sectional study was to assess differences in reporting between information self-reported by nursing homes and Medicare claims by calculating rates of reporting for antipsychotic drug use and the diagnosis of schizophrenia, Tourette's syndrome, and Huntington's disease. We also examined the within-facility characteristics associated with better reporting and investigated the interaction between the reporting of antipsychotic drug use and reporting of a qualifying condition, by data source.

## Methods

### Data source

The study used the MDS 3.0 from January 1, 2018, to December 31, 2019, which contains self-reported clinical assessments of nursing home residents’ health, physical functioning, mental status, and general wellbeing. MDS assessments are required for residents upon admission to the nursing facility, quarterly, annually, and at discharge, or if there are any significant changes to the resident's condition.^12^ We focused on MDS item N0410A, which reports the number of days (0–7 d) that a resident received antipsychotic medication in the past 7 days or since admission/re-entry if less than 7 days.^13^

We also used Medicare claims from January 1, 2017, to December 31, 2019. Medicare Parts A and B contain diagnosis information for beneficiaries enrolled in Traditional Medicare (TM), and Part C Medicare Advantage (MA) encounter data contain diagnosis information for beneficiaries enrolled in MA. Medicare Part D was used to collect information about the type, date, and duration of drugs dispensed. Part D claims availability was limited to a 20% random sample of Medicare beneficiaries. We used enrollment and demographic information from the Medicare beneficiary summary files to obtain patient-level characteristics (age, sex, race/ethnicity, dual-eligible status, MA enrollment) for the 20% analytic sample. Our claims data include an extra year of data relative to the MDS to provide a 1-year pre-period to identify the diagnosis of conditions in the claims (eg, 2018 MDS data required 2017 claims data to look-back 1 year). Facility-level characteristics and information about a facility's star rating were obtained from CMS’ Nursing Home Care Compare website.^14^

This study was approved by the institutional review board of Harvard Medical School.

### Study sample

We identified Medicare beneficiaries in a random 20% sample aged 65 and older who were enrolled in Medicare (TM or MA) for a continuous 12 months in each calendar year. Medicare beneficiaries identified in the claims were linked to person-level nursing home assessments in the MDS by quarter. Because prescription drugs are covered under Medicare Part A for short-stay residents, we limited the sample to long-stay residents who resided in a nursing home for longer than 90 days.^15–17^

### Outcomes and variable construction

#### Identification of antipsychotic drugs

To identify reporting inconsistencies between the MDS and Medicare claims, we created lookback windows for antipsychotic drug use in each dataset. In the Medicare Part D claims, we identified antipsychotic drugs following prior work from CMS on antipsychotic use in Part D enrollees, which included both typical (first-generation) and atypical (second-generation) antipsychotics^18,19^ (Table A1 in the Supplement). In the MDS, we created a 30-day lookback window from the date on which antipsychotic use was reported on the MDS assessment. We then created a window of dates to capture antipsychotic prescriptions in the Part D data, beginning with the date a prescription was filled and ending with a date based on the numbers of days supplied (eg, 30 d, 90 d, etc.). We checked whether the date of the antipsychotic prescription in the Part D claims (based on the window using the drug fill date and supply end date) was within the MDS assessment date and 30-day lookback date.

#### Identification of qualifying conditions

Because schizophrenia, Tourette's syndrome, and Huntington's disease are the 3 conditions that are considered appropriate for the receipt of antipsychotics in CMS’ quality star rating calculation, we used diagnosis, procedure, or adverse event International Classification of Diseases, 10th Revision (ICD-10), codes (Table A2 in the Supplement) to identify these diagnoses in Medicare inpatient and outpatient claims for fee-for service beneficiaries (Part A, B) and Medicare advantage beneficiaries (Part C) based on prior CMS work linking MDS items to ICD codes.^18^ In the MDS, we used items I6000, I5350, and I5250, which indicated whether a resident had an active diagnosis of schizophrenia, Tourette's syndrome, or Huntington's disease in the previous 7 days. We checked whether there was a corresponding Medicare diagnosis claim in the preceding 4 quarters from the MDS assessment.

### Outcome measures

To assess the reporting of antipsychotic drug use and qualifying conditions, we created measures to capture the reporting rate between the MDS and Medicare claims for antipsychotics and each condition. The antipsychotic drug reporting variable captured whether there was both an indicator for antipsychotics received in the MDS and a prescription for antipsychotics in the Part D claims. Reporting for antipsychotics was recorded if the date of the antipsychotic fill in the Part D claims occurred prior to the MDS assessment and the 30-day lookback period was within the possible range of dates for the antipsychotic prescription supply (eg, between the fill date and supply date) and is conditional on antipsychotics reported in the Part D claims.

Because we were interested in whether residents acquired conditions in the MDS that were unsupported by a corresponding Medicare claims diagnosis, the condition reporting variables were conditional on reporting in the MDS. The condition reporting rate captured whether there was both an indicator for an MDS diagnosis of the condition and a corresponding claims diagnosis in the preceding 4 quarters from the MDS diagnosis, conditional on reporting in the MDS. Due to small counts of residents with Tourette's syndrome or Huntington's disease, a measure was also created to capture whether any one of the conditions (schizophrenia, Tourette's, or Huntington's) was reported in the MDS and claims, conditional on reporting in the MDS.

### Statistical analysis

We assessed reporting between the MDS and Medicare claims by calculating rates of reporting for antipsychotic drug use and the diagnosis of schizophrenia, Tourette's syndrome, Huntington's disease, or any 1 of the 3 conditions across all patient-quarters in our data.

To estimate the characteristics associated with greater odds of reporting the drug or condition (schizophrenia or any condition) in the MDS, we used multivariable logistic models with quarter and nursing home fixed effects to understand which factors were associated with variation in reporting rates within facilities. The model included patient-level variables for age (65–74, 75–84, 85+ y), sex, enrollment in MA, dual-eligibility status (eligible for both Medicare and Medicaid), diagnoses of Alzheimer's disease and related dementias (ADRD), and race (White, Black, Asian, Hispanic, unknown, or other). After observing a strong association between race and dual status, we included an interaction of these variables in the model. We also tested a model that included an indicator for receiving a Medicare Part D LIS for prescription drug coverage instead of a dual-eligible indicator.

Finally, we investigated the relationship between antipsychotic drug use and condition reporting by focusing on those with antipsychotic drugs reported in both the MDS and Medicare claims. We disaggregated the antipsychotic reporting rate variable by the share that also reported the condition in both the MDS and Medicare claims, the condition reported only in the MDS or only in Medicare claims, or no condition that qualified for exclusion from CMS’ quality measure reported.

Statistical tests were 2-tailed and *P* values <.05 were considered statistically significant. All statistical analyses were performed using Stata/MP, version 16.1 (StataCorp LLC).

### Limitations

First, this study was limited to nursing home residents in a 20% Medicare sample due to data availability. Although the sample is random, some differences may be present in our 20% Medicare analytic sample compared with the full sample of Medicare beneficiaries. Additionally, we included MA beneficiaries in our study sample, but issues with the completeness of Part C encounter data^20^ may limit the identification of diagnoses and medication use for this group. Second, we used a 90-day cutoff to identify long-stay nursing home residents in the MDS, but we may capture a few short-stayers using this method. Third, we identified antipsychotic drugs in the Part D claims following previous work by CMS, but it is not possible to know for certain the list of antipsychotic drugs a nursing home uses to record the number of days an antipsychotic was received or if this list is consistent across nursing homes. Fourth, MDS assessments are self-reported and based on a 7-day lookback period, so this measure captures antipsychotic use at a single point in time and some variation may exist in the completeness and accuracy. More specifically, because MDS assessment varies in frequency (eg, quarterly, annually, as needed), there may be antipsychotic drug use that occurred between MDS assessments that are not captured in the 7-day lookback period of the MDS item. There may also be differences in measurement error between facilities that use their electronic health record (EHR) to auto-populate the MDS antipsychotic item compared with facilities that manually input the information. Finally, due to sample size limitations of the population of residents with Tourette's syndrome or Huntington's disease, we were not able to analyze reporting rates for these items separately and instead used a combined measure for the diagnosis of any condition. Although schizophrenia diagnoses comprised 98% of the any-condition group, we included the any-condition outcome in our analyses to provide alignment with the qualifying conditions that CMS considers for its star rating measure.

## Results

### Sample characteristics

The study sample consisted of 238 778 patients (1 149 583 patient-quarters) residing in 15 684 US nursing homes. The overall sample was mostly female (69.2%), dual-eligible (75.9%), diagnosed with ADRD (60.6%), and was majority White (80.2%), with a mean age of 83 years (Table 1). Residents with antipsychotics reported in both the MDS and Medicare claims were slightly younger on average (79.1 y) and more likely to be dual eligible (85%) or have ADRD (69.7%). Those with schizophrenia or any condition (schizophrenia, Tourette's syndrome, Huntington's disease) reported in both the MDS and claims were more likely to be younger (74.9 y), less likely to be covered by MA (16.4% and 16.5%, respectively), more likely to be dual eligible (91.9% and 91.6%, respectively), or have ADRD (53%), and less likely to be White (74.2% and 74.4%, respectively) compared with the overall sample.

**Table 1.** Sample characteristics.

	Full sample	Antipsychotic reporting	Schizophrenia reporting	Any condition reporting
No.	1 149 583	186 005	51 615	52 672
Resident characteristics				
Age, y	82.95	79.09	74.90	74.88
Sex (female)	69.18%	67.42%	60.83%	60.84%
Medicare Advantage	24.69%	23.3%	16.38%	16.48%
Dual eligible	75.89%	84.95%	91.91%	91.61%
ADRD	60.64%	69.74%	53%	52.94%
Race				
Other or unknown^a^	1.99%	1.86%	1.9%	1.93%
White	80.21%	80.82%	74.18%	74.4%
Black	13.89%	14.17%	20.98%	20.75%
Asian	1.81%	1.08%	0.9%	0.89%
Hispanic	2.11%	2.08%	2.04%	2.03%
Facility characteristics				
Bed size	141.59	145.64	147.74	147.6
Number of residents	119.54	122.47	123.22	123.08
Share Medicaid residents	63.20%	67.49%	71.87%	71.75%
Ownership				
Nonprofit	23.15%	18.68%	11.27%	11.59%
Government	7.48%	7.03%	6.73%	6.7%
For-profit	69.36%	74.29%	82%	81.71%
Overall rating				
1–2 Stars	35.44%	40.71%	47.52%	47.4%
3 Stars	17.75%	18.22%	18.84%	18.77%
4–5 Stars	46.81%	41.07%	33.64%	33.83%
Quality rating				
1–2 Stars	16.88%	20.47%	21.28%	21.23%
3 Stars	17.32%	18.65%	18.64%	18.62%
4–5 Stars	65.79%	60.88%	60.08%	60.16%
Census region				
West	12.65%	11.56%	13.32%	13.44%
Northeast	26.46%	27.09%	25.11%	25.02%
Midwest	27.20%	27.19%	27.44%	27.56%
South	33.70%	34.17%	34.13%	33.98%

Abbreviation: ADRD, Alzheimer's disease and related dementias.

Data are presented as means or percentages. Source: Authors’ analysis of Medicare Claims and Minimum Data Set 3.0, 2018–2019.

a American Indian, Alaska native, native Hawaiian, or Pacific Islander.

### Overall rates of reporting

Across the study sample, MDS resident assessments reported antipsychotic drug use in 211 745 patient-quarters or only 87.8% of the antipsychotic use reported according to Part D claims (Table 2). Additionally, only 54.8% of the patient-quarters with a diagnosis of schizophrenia in the MDS had a corresponding diagnosis identified in the claims data. We also identified reporting rates in the MDS of 46.5% for Tourette's syndrome, 72.4% for Huntington's disease, and 55.0% for any 1 of the 3 conditions relative to reporting in the claims data.

**Table 2.** Overall reporting rates of antipsychotic drug use and qualifying conditions (at the patient-quarter level).

	Antipsychotics	Schizophrenia	Tourette's	Huntington's	Any condition^a^
Any drug/diagnosis in MDS	216 385	94 275	316	1369	95 788
Any drug/diagnosis in Medicare claims	211 745	60 450	192	1138	61 638
In both the MDS and Medicare claims^b^	186 005	51 615	147	991	52 672
Reporting rate,^c^ %	87.84%	54.75%	46.52%	72.39%	54.99%

Abbreviation: MDS, Minimum Data Set.

Source: Authors’ analysis of Medicare Claims and Minimum Data Set 3.0, 2018–2019.

a Any condition includes diagnosis of schizophrenia, Tourette's syndrome, or Huntington's disease.

b Antipsychotic drugs were identified within the previous 30-days in Medicare Part D claims and diagnoses were identified within the previous 4 quarters in Medicare claims relative to the MDS assessment date.

c Antipsychotic reporting rate variable was conditional on being reported in the Part D claims; diagnosis outcomes were conditional on being reported in the MDS.

### Differences in reporting rates

**Figure 1.**
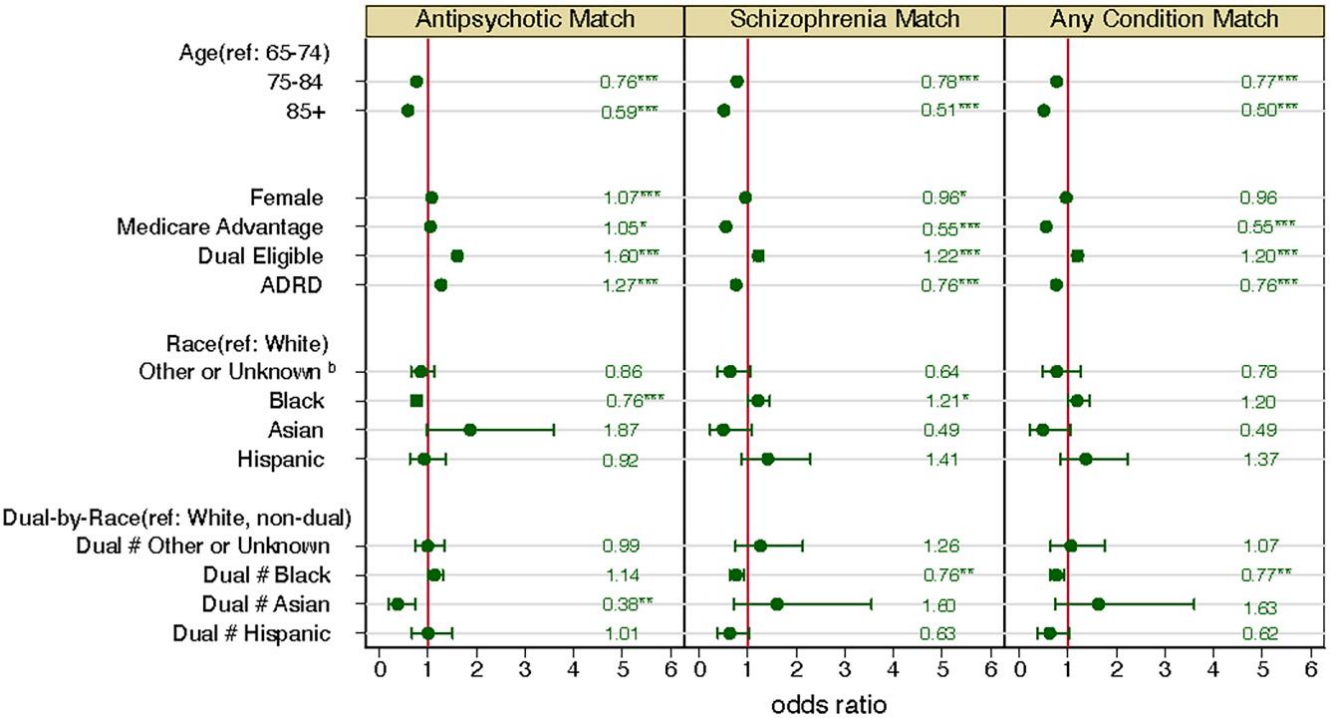
Multivariable logistic regression results for antipsychotic reporting, schizophrenia reporting, or any condition reporting^a^. Coefficient estimates are presented for the odds ratio from a multivariable logistic regression with quarter and nursing home fixed effects with robust standard errors. Bars represent 95% CIs. *P < .05; **P < .01; *** *P* <0.001. ^a^Antipsychotic reporting rate variable was conditional on being reported in the Part D data for the antipsychotic outcome; diagnosis outcomes (schizophrenia or any condition) were conditional on being reported in the Minimum Data Set 3.0. ^b^American Indian, Alaska native, native Hawaiian, or Pacific Islander. Abbreviations: ADRD, Alzheimer's disease and related dementias; ref, reference. Source: Authors’ analysis of Medicare Claims and Minimum Data Set 3.0, 2018–2019.

In our multivariable logistic models, we found that dual-eligibility status was associated with 1.6 times higher odds of reporting in the MDS and Medicare claims for antipsychotic drugs conditional on reporting in the claims data and 1.2 times higher odds of reporting schizophrenia or any condition, conditional on reporting in the MDS compared with non–dual-eligibles (Figure 1). Black residents had a 0.8 times lower odds of reporting antipsychotic use compared with White residents. Being insured by MA or having ADRD was associated with higher odds of reporting for antipsychotics, but lower odds of reporting for schizophrenia or any condition. These results were consistent when including an indicator for residents receiving a Part D LIS instead of an indicator for dual-eligibility status (Table A3).

### Reporting rates between antipsychotic use and qualifying conditions

**Figure 2.**
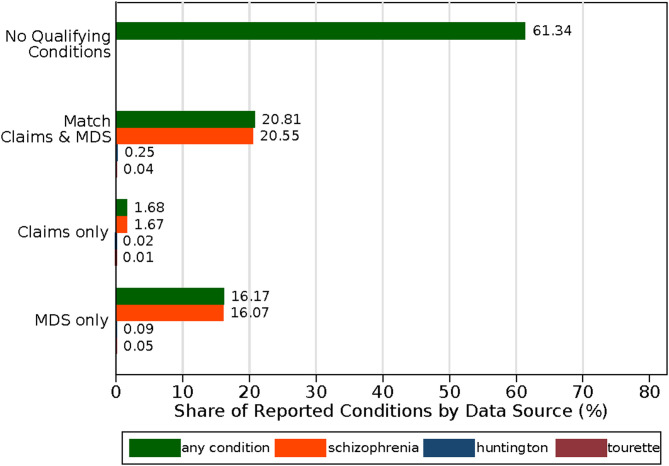
Reporting of qualifying conditions by data source, conditional on antipsychotic reporting. Conditional on antipsychotic drug use reported in the MDS and claims (87.8% reporting rate), each bar disaggregates the share of drug use that also had each condition reported by data source (eg, both MDS and claims, claims only, MDS only). “No Qualifying Conditions” refers to the share of antipsychotic use in the MDS and claims that did not report any condition (schizophrenia, Huntington's disease, or Tourette's syndrome) in the MDS or claims data. Abbreviation: MDS, Minimum Data Set. Source: Authors’ analysis of Medicare Claims and Minimum Data Set 3.0, 2018–2019.

Among the 87.8% of patient-quarters with antipsychotics recorded in the MDS and Medicare claims, 61.3% of these instances did not have a corresponding diagnosis in the MDS or claims for 1 of the 3 qualifying conditions (Figure 2). A total of 20.8% had a diagnosis in the MDS and claims data for any qualifying condition (20.6% reporting rate for schizophrenia and <0.5% for Tourette's and Huntington's combined). An additional 16.2% had a condition reported in the MDS only (16.1% reported an MDS schizophrenia diagnosis and <0.5% reported an MDS Tourette's or Huntington's diagnosis), while only 1.7% had a condition reported in Medicare claims only.

## Discussion

We identified underreporting of antipsychotic drugs but overreporting of the conditions that qualify for an exemption from CMS’ star rating measure. Our reporting estimates were consistent with prior work by the OIG and others on the overall trends of psychotropic drug use and reporting, which found higher drug use in nursing homes with certain characteristics, such as those with higher shares of residents receiving LIS^9,11^ or facilities with more Medicaid residents, smaller size, and fewer registered nursing hours.^21^ Our work builds upon the OIG investigations by identifying characteristics within facilities that were associated with greater reporting. We found a strong association by dual-eligibility status and race, as duals had higher odds of reporting antipsychotic drug use and the qualifying conditions, while Black race was associated with lower odds of antipsychotic reporting. Our results using nationally representative claims data build upon prior evidence from an international study^22^ and a single-state analysis using Medicaid claims^23^ identifying an association between reductions in potentially inappropriate antipsychotic drug use and corresponding increases in the diagnosis of qualifying conditions.

Our work is also consistent with prior work on the reporting rates of pressure ulcers^8^ and falls^7^ in the MDS and Medicare claims, which found that certain resident characteristics were associated with a better reporting rate. We identified much higher rates for antipsychotic drug use relative to the reporting rate found for falls or pressure ulcers, which may be attributed to several CMS initiatives and attention directed at this issue.^2,24^ It could also be that drug information captured in an MDS assessment may be directly populated from a nursing home's EHR system, although there is variability in EHR use and functionality as a result of slow adoption or differences in facility resources.^25^

In our study, over half of the sample with antipsychotic drug use and diagnosis of a qualifying condition identified in both the MDS and claims were also diagnosed with ADRD. The CMS’ National Partnership was targeted specifically at reducing antipsychotic drug use in residents with ADRD, with prior literature highlighting declines in antipsychotic use generally^26,27^ as well as for those with ADRD before and after the National Partnership.^28,29^ While CMS was particularly concerned about potentially inappropriate antipsychotic use among residents with dementia, other work has established declines in antipsychotic use among those with one of the qualifying conditions, for whom this medication use may be appropriate and necessary.^28^ Recent pilot audits conducted by CMS have also noted that residents may have behaviors noted in their medical records that are related to dementia, and not schizophrenia.^30^ This underscores the need for the accurate reporting of diagnoses to understand whether antipsychotic use is appropriate, as it may be for residents with one of the qualifying conditions, or if it is potentially inappropriate, as it may be for residents with ADRD.

We also noted significant effects by race and dual-eligibility status. Prior literature has established that dual-eligibility status and Black race were associated with receiving care from lower-quality facilities.^31,32^ Although the direction of antipsychotic use by race and dual status may differ between facilities, as 1 analysis found that higher shares of Black residents were associated with lower rates of inappropriate antipsychotic use while higher shares of Medicaid residents were associated with higher rates of use.^33^ These findings may help explain the difference in direction of the results identified in our within-facility analysis.

More specifically, we identified that Black race was associated with lower odds of reporting for antipsychotics. Prior work has highlighted disparities between Black and White nursing home residents, with higher rates of antipsychotic use^34,35^ and higher rates of schizophrenia diagnoses among Black residents.^36^ Additionally, recent work has highlighted an interaction between race and ADRD status, as Black residents with ADRD were more likely to have a schizophrenia diagnosis documented on their MDS assessments after the National Partnership.^37^ However, despite higher baseline rates of drug use and diagnoses of schizophrenia, Black residents experience lower reporting rates as they are less likely to have their antipsychotic drug use reported in the MDS, conditional on reporting in the claims. Policies that target reductions in antipsychotic use in vulnerable populations may have potential unintended consequences that reinforce disparities in care and may contribute to the observed variation in reporting rates experienced by Black residents compared with residents of other races.

The effects of dual-eligibility status were larger compared with the effect by race, which is consistent with other work studying post–acute-care transitions, which found differences by dual status that were more pronounced compared with racial/ethnic differences.^38^ Medicare beneficiaries who are also eligible to receive Medicaid benefits often have lower income and fewer resources, more complex clinical and social needs, higher health expenditures, and experience fragmented care coordination.^39^ Although a long-stay resident's drug use is covered by Medicare Part D, Medicaid covers long-term nursing home care. As a result, the higher reporting observed for dual-eligibles may be the result of oversight from 2 separate programs (both the federal Medicare program and state Medicaid programs). Although individual states have flexibility about who and what benefits to cover under their Medicaid programs, our results were robust when using an indicator for the Medicare Part D LIS.

Our work is particularly timely given that the Biden administration^40^ began conducting targeted off-site audits in select nursing homes in January 2023. Nursing homes will be audited to assess whether they have accurately coded residents with schizophrenia. Facilities will have their quality star downgraded to 1 star by CMS if there is inaccurate coding, with survey citations for deficiencies displayed on the Care Compare website.^40^ In particular, CMS is concerned with erroneous coding of diagnoses, as pilot audits by CMS have found that some residents lack comprehensive psychiatric evaluations or behavior documentation.^30^ Additionally, most individuals are diagnosed with schizophrenia by age 40 years and late-onset is rare.^41^ Our findings of schizophrenia diagnoses in the MDS for our Medicare population aged 65+ that were unsupported by a corresponding claims diagnosis underscore the need to uncover whether these diagnoses were due to “phony diagnoses” or other factors driving changes in diagnosis coding, such as increased attention to the assessment of residents with dementia after CMS’ National Partnership^42^ or improvement in documentation to support medication use.^43^

Our work suggests that almost one-fifth of antipsychotic drug use in residents with a diagnosis of a qualifying condition in the MDS were unsupported by a corresponding Medicare claims diagnosis. Although such use is currently deemed as appropriate and excluded from the quality star rating measure under CMS’ current standards for antipsychotic reporting, this use would be classified as inappropriate if diagnoses of schizophrenia, Tourette's syndrome, or Huntington's disease in the MDS were validated against Medicare claims diagnoses. While appropriate antipsychotic use is one of many factors that contribute to a facility's CMS quality star rating, the implications of our results suggest that many nursing homes might experience a lower quality star rating if these discrepancies were accounted for.

## Conclusion

In this cross-sectional study examining the reporting rates between data reported in the MDS and Medicare claims, we identified underreporting for the use of antipsychotics and overreporting of qualifying conditions associated with appropriate antipsychotic use. Our findings show that many nursing home residents who received antipsychotic drugs do not also have one of the qualifying conditions in both the MDS and Medicare claims to be excluded from CMS’ quality star measure. Future work should continue to investigate whether this antipsychotic use is appropriate and if nursing homes may be diagnosing residents with certain conditions to avoid penalization by CMS.

## Supplementary Material

qxad022_Supplementary_Data
