# Supplementary material for: Nursing homes underreport antipsychotic use but overreport diagnoses qualifying for appropriate use
Source: Health Aff Sch. 2023 Jun 20;1(2):qxad022. doi: 10.1093/haschl/qxad022 (PMC10846688; doi:10.1093/haschl/qxad022)
Supplement: qxad022_Supplementary_Data [file qxad022_Supplementary_Data.zip › antipsychotics_appendix_revision_clean_05_22_23.docx]

**Appendix**

**Exhibit A1** List of Antipsychotic drugs (table)

| **drug name** | **type** |
| --- | --- |
| Aripiprazole | atypical |
| Asenapine | atypical |
| Chlorpromazine | conventional |
| Clozapine | atypical |
| Fluphenazine | conventional |
| Haloperidol | conventional |
| Iloperidone | atypical |
| Loxapine | conventional |
| Lurasidone | atypical |
| Olanzapine | atypical |
| Paliperidone | atypical |
| Perphenazine | conventional |
| Pimozide | conventional |
| Quetiapine | atypical |
| Risperidone | atypical |
| Thioridazine | conventional |
| Thiothixene | conventional |
| Trifluoperazine | conventional |
| Ziprasidone | atypical |

**Exhibit A2** List of conditions identified in claims and MDS (table)

| **Condition** | **Claims (ICD-10 codes)** | **MDS** |
| --- | --- | --- |
| **Schizophrenia** (schizoaffective and schizopheniform disorders) | [F20 Schizophrenia](https://www.icd10data.com/ICD10CM/Codes/F01-F99/F20-F29/F20-/F20) | (*I6000_SCHZOPRNIA_CD*)  Indicator for whether the resident had an active diagnosis of schizophrenia (schizoaffective and schizopheniform disorders) in the last seven days. |
|  | [F20.0 Paranoid schizophrenia](https://www.icd10data.com/ICD10CM/Codes/F01-F99/F20-F29/F20-/F20.0) |  |
|  | [F20.1 Disorganized schizophrenia](https://www.icd10data.com/ICD10CM/Codes/F01-F99/F20-F29/F20-/F20.1) |  |
|  | [F20.2 Catatonic schizophrenia](https://www.icd10data.com/ICD10CM/Codes/F01-F99/F20-F29/F20-/F20.2) |  |
|  | [F20.3 Undifferentiated schizophrenia](https://www.icd10data.com/ICD10CM/Codes/F01-F99/F20-F29/F20-/F20.3) |  |
|  | [F20.5 Residual schizophrenia](https://www.icd10data.com/ICD10CM/Codes/F01-F99/F20-F29/F20-/F20.5) |  |
|  | [F20.8 Other schizophrenia](https://www.icd10data.com/ICD10CM/Codes/F01-F99/F20-F29/F20-/F20.8) |  |
|  | [F20.81 Schizophreniform disorder](https://www.icd10data.com/ICD10CM/Codes/F01-F99/F20-F29/F20-/F20.81) |  |
|  | [F20.89 Other schizophrenia](https://www.icd10data.com/ICD10CM/Codes/F01-F99/F20-F29/F20-/F20.89) |  |
|  | [F20.9 Schizophrenia, unspecified](https://www.icd10data.com/ICD10CM/Codes/F01-F99/F20-F29/F20-/F20.9) |  |
|  | F25.0 Schizoaffective disorder, bipolar type |  |
|  | F25.1 Schizoaffective disorder, depressive type |  |
|  | F25.8 Other schizoaffective disorders |  |
|  | F25.9 Schizoaffective disorder, unspecified |  |
| **Tourette’s syndrome** | F95.2 | (*I5350_TOURT_CD*)  Indicator for whether the resident had an active diagnosis of Tourette's Syndrome in the last seven days. |
| **Huntington's disease** | G10 | (*I5250_HNTGTN_CD*)  Indicator for whether the resident had an active diagnosis of Huntington's Disease in the last seven days. |

NOTES MDS is Minimum Data Set.

**Exhibit A3** Multivariable Logistic Regression Results with Part D low-income subsidy (table)

|  | | | | | | |
| --- | --- | --- | --- | --- | --- | --- |
|  | **Match Antipsychotics ^a^** | | **Match Schizophrenia ^b^** | | **Match Any Condition ^b^** | |
|  | Odds ratio (95% CI) | | Odds ratio (95% CI) | | Odds ratio (95% CI) | |
| **Age**  **(ref: 65-74)** |  |  |  |  |  |  |
| **75-84** | 0.764*** | (0.73, 0.79) | 0.780*** | (0.75, 0.82) | 0.769*** | (0.74, 0.80) |
| **85+** | 0.589*** | (0.56, 0.62) | 0.515*** | (0.48, 0.55) | 0.508*** | (0.48, 0.54) |
| **Female** | 1.067*** | (1.03, 1.11) | 0.951* | (0.91, 0.99) | 0.959 | (0.92, 1.0) |
| **Medicare Advantage** | 1.049* | (1.01, 1.09) | 0.546*** | (0.52, 0.58) | 0.549*** | (0.52, 0.58) |
| **Part D LIS** | 1.695*** | (1.61, 1.78) | 1.490*** | (1.36, 1.63) | 1.450*** | (1.32, 1.58) |
| **ADRD** | 1.271*** | (1.22, 1.32) | 0.757*** | (0.72, 0.79) | 0.759*** | (0.73, 0.79) |
| **Race (ref: White)** |  |  |  |  |  |  |
| **Other or Unknown** | 0.804 | (0.60, 1.08) | 0.675 | (0.39, 1.17) | 0.773 | (0.45, 1.32) |
| **Black** | 0.762*** | (0.66, 0.89) | 1.019 | (0.83, 1.25) | 0.999 | (0.81, 1.22) |
| **Asian** | 1.447 | (0.73, 2.86) | 0.412 | (0.16, 1.07) | 0.402 | (0.15, 1.04) |
| **Hispanic** | 0.755 | (0.44, 1.31) | 1.114 | (0.61, 2.03) | 1.087 | (0.59, 1.98) |
| **LIS*race (ref: white, non-dual)** |  |  |  |  |  |  |
| **Other or Unknown** | 1.069 | (0.78, 1.47) | 1.181 | (0.67, 2.09) | 1.068 | (0.61, 1.86) |
| **Black** | 1.137 | (0.97, 1.33) | 0.909 | (0.74, 1.12) | 0.924 | (0.75, 1.14) |
| **Asian** | 0.504 | (0.25, 1.01) | 1.909 | (0.73, 5.02) | 1.957 | (0.74, 5.15) |
| **Hispanic** | 1.229 | (0.70, 2.14) | 0.802 | (0.43, 1.48) | 0.795 | (0.43, 1.47) |

SOURCE Authors’ analysis of Medicare Claims and Minimum Data Set 3.0, 2018-2019. NOTES LIS is low-income subsidy. ADRD is Alzheimer’s disease and related dementias. 95% CI is 95% confidence interval. Logistic regressions include quarter and nursing home fixed effects and robust standard errors. * p<.05; ** p<.01; *** p<.001

^a^ conditional on reporting the drug in Medicare Part D

^b^ conditional on reporting the diagnosis in the MDS
